# Ectopic thyroid with benign and malignant findings: A case series

**DOI:** 10.1016/j.ijscr.2019.11.011

**Published:** 2019-11-18

**Authors:** J. Lukáš, J. Drábek, D. Lukáš, I. Zemanová, A. Rulseh

**Affiliations:** a Department of Otolaryngology and Head and Neck Surgery, Na Homolce Hospital, Prague, Czech Republic; b Institute of Molecular and Translational Medicine, Faculty of Medicine and Dentistry, Palacky University Olomouc, Czech Republic; c Department of Surgery, 3rd Faculty of Medicine, Charles University and University Hospital in Prague, Czech Republic; d Department of Pathology, Na Homolce Hospital, Prague, Czech Republic; e Department of Radiology, Na Homolce Hospital, Prague, Czech Republic

**Keywords:** Lingual thyroid, Thyroglossal duct carcinoma, Cribriform-morular papillary carcinoma, Ectopia

## Abstract

**Introduction:**

Ectopia is the most common sporadically occurring thyroid heterotopy. We present three cases of ectopic thyroid tissue with compression of the upper aerodigestive tract. The first case involved ectopic thyroid tissue in the lingual area of a 60-year-old male with dysphagia, swelling at the base of the tongue, and stomatolalia. The second case was a 66-year-old female with papillary thyroid carcinoma (PTC) in a thyroglossal duct cyst. The third patient was a 50-year-old female with aberrant thyroid tissue in the right submandibular region, with a cribriform-morular variant of PTC (CMV-PTC).

**Methods:**

After resecting the heterotopic tissue and verifying the presence of PTC, the second and third cases underwent total thyroidectomy, and the third patient also underwent radioactive iodine ablation (RAI). Postoperative athyreosis was compensated by permanent levothyroxine substitution.

**Results:**

The diagnosis of ectopic thyroid tissue is challenging. Clinical examination together with imaging methods play a key role, especially postoperative histological examination along with scintigraphy and single photon emission computed tomography (SPECT). Ultrasonography should be used to exclude normally localized thyroid tissue and to distinguish other tumorous diseases. In the pre-operative examination, ultrasound-guided fine-needle aspiration biopsy (US-FNAB) often results in technically-difficult sampling and non-diagnostic cytology.

**Conclusion:**

Resection is the most suitable therapy for clinical symptoms of a foreign body in the upper aerodigestive tract and inflammatory complications; total thyroidectomy follows in case of malignant transformation. Thyroid heterotopy is a rare pathological condition, yet it should be taken into consideration during differential diagnosis of tumorous oropharyngeal and neck lesions.

## Highlights

• Thyroid heterotopy can be a cause of tumorous oropharyngeal and neck lesions.
• We present three cases of ectopic thyroid tissue with compression of the upper aerodigestive tract in the lingual area, thyroglossal duct cyst, and right submandibular region.
• We describe clinical and imaging examinations for diagnosis and treatment.

## 1 Introduction

The thyroid gland begins to form in the third week of foetal life from the primitive pharynx and neural crest as an endodermal invagination of the tongue at the site of the foramen cecum. At approximately the 7th week of fetal life, it descends via the thyroglossal duct to its final location, anteriorly to the pre-trachea and larynx [1,2]. The most common disorder of thyroid dysgenesis is its failure to descend from the foramen cecum and the persistence of the thyroglossal duct, reported in approximately 7 % of cases, which results in congenital predisposition for the formation of a thyroglossal duct cyst, thyroglossal duct fistula, or rarely, carcinoma of the thyroglossal duct remnants [6,7]. Lingual location of thyroid tissue counts for 48–61 % of all thyroid dysgeneses [3,4]. Its prevalence reaches 1 per 100,000–300,000 persons [4], occurring more frequently in females (75–80 %) [4,5]. In rare cases, ectopic tissue can be located outside the midline neck, in submandibular region, surrounding the cervical lymph nodes, and as an aberrant thyroid due to the failure of the involution of lateral buds of the 4th brachial pouch [4]. The most common clinical manifestations of ectopic thyroid (in 80–85 % cases) include globus pharyngeus, dysphagia and congenital hypothyreosis [[1], [2], [3], [4]]. In about 1 % cases, ectopic thyroid tissue is transformed into thyroid carcinoma [3]. In 70–90 % of cases, it is the only thyroid tissue present [1], usually in the absence of an orthotopic thyroid [5]. Radiological imaging modalities, such as ultrasound, CT scan (CT), magnetic resonance imaging (MRI) and scintigraphy play a key role in diagnosing ectopia [1,2].

## 2 Methods

Eligibility criterion was presence of ectopic thyroid tissue with compression of the upper aerodigestive tract. This work has been reported in accordance with the PROCESS criteria [8]. All procedures were performed by a single investigator (JL) with 25 years experience.

### 2.1 Case 1

A 60-year-old non-smoker male with increasing oropharyngeal dysphagia and progressing stomatolalia in the past six months was referred for an ENT examination. Hypothyroidism was compensated with 150 μg of levothyroxine, given daily.

### 2.2 Case 2

A 66-year-old non-smoker female was admitted to our ENT department with a tumor mass in the submental region that she reported having for over 5 years. In the last six months, she observed its accelerated growth, accompanied by a globus sensation.

### 2.3 Case 3

A 50-year-old non-smoker female was admitted to our ENT department for extirpation and histological examination of a tumor mass in the submental space, to the right of the midline. The patient had noticed resistance for approximately 7 years; growing progressively in the last 4 months, accompanied by a pressure sensation on the right side of the neck, especially during swallowing.

## 3 Results

### 3.1 Results of case 1

**Fig. 1:**
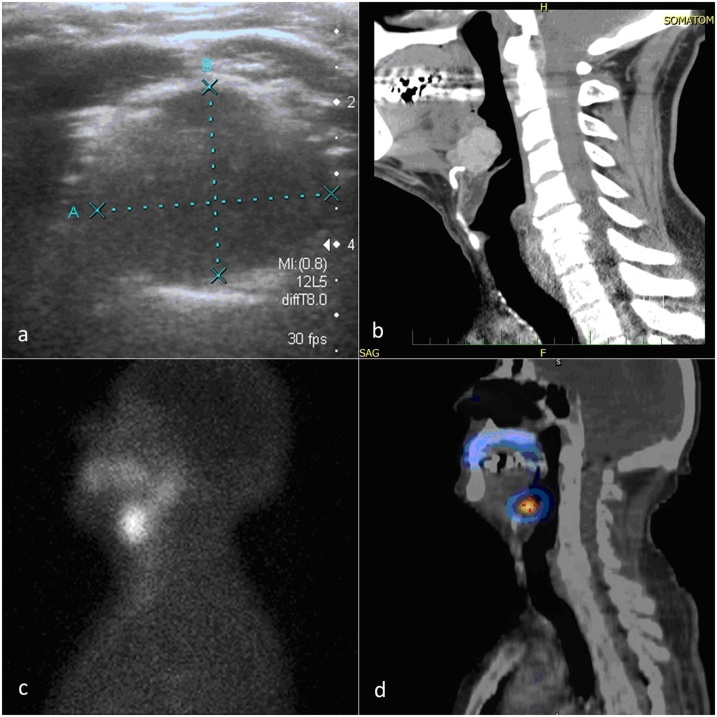
**Imaging modalities** a) bounded, solid hypoechogenic, slightly vascularised formation 19 × 17 × 9 mm, b) CT scan of lateral projection of heterotopic thyroid tissue in the root of the tongue, without the presence of the thyroid in the usual neck position, c) scintigraphy and, d) SPECT (single photon emission computed tomography) radiopharmaceuticals (^99m^Tc-pertechnetate) in the root of the tongue and confirmed the absence of thyroid tissue in its usual location in the anterior neck.

**Fig. 2:**
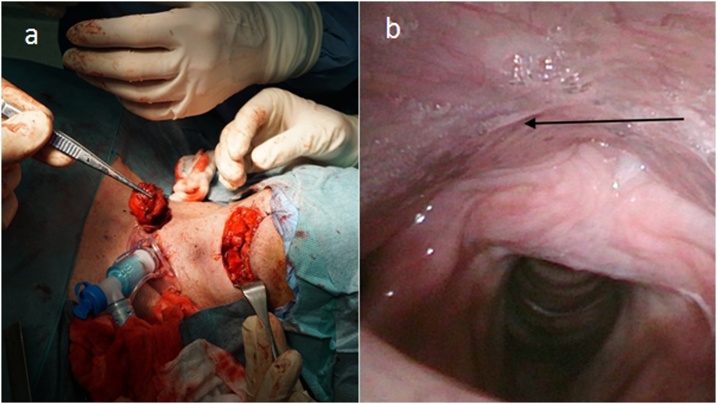
**Operational approach** a) temporary tracheostomy and external surgical approach-transhyoid pharyngotomy and, lingual thyroid in tweezers, b) arrow - scar in hypopharynx, 6 months after the surgery.

Epipharyngoscopy revealed a mass in the base of the tongue and incisional biopsy discounted malignancy. FNAB was not used. Neck sonography did not detect thyroid tissue in its usual location. Computed tomography (CT) revealed a mass (31 × 31 × 27 mm) at the base of the tongue, protruding into the right vallecula. Scintigraphy confirmed an accumulation of 99mTc-pertechnetate in the root of the tongue and the absence of thyroid tissue on the anterior of the neck (Fig. 1A–D). The swelling gradually increased in size and growing dysphagia and stomatolalia resulted in resection (external approach), transhyoid pharyngotomy and temporary tracheostomy (Fig. 2). The surgery started with tracheostomy to prevent suffocation due to postoperative edema of the root of the tongue, to eliminate endotracheal intubation from the hypopharynx and to increase visibility and manipulability in the surgical area. The postoperative period was without complications. Histology verified unencapsulated thyroid tissue growing into the surrounding striated muscle and adipose tissue, without malignant structures. Patient follow-up is once a year by us and twice a year by endocrinologist. He is without complications.

### 3.2 Results of case 2

**Fig. 3:**
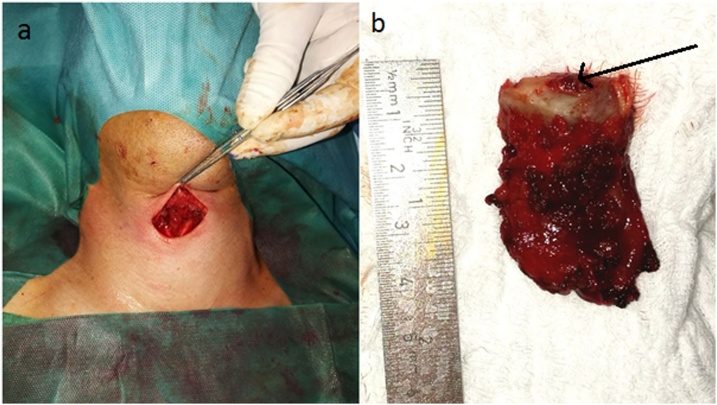
**Operational approach** a) situation after the resection of the cyst b) extirpated cyst with the hyoid bone and thyroglossal duct (arrow).

Clinical examination revealed a cystic formation superior to the thyroid cartilage of 30 × 17 × 10 mm, reaching the hyoid bone. Upon palpation, neither the thyroid gland nor the cervical lymph nodes were enlarged. Biochemically, the patient was euthyroid with normocalcemia. Ultrasonography confirmed a well-differentiated vascularised multilocular cyst in the midline of the neck, between hyoid bone and the superior edge of thyroid cartilage. The left lobe contained three calcified nodules <15 mm. FNAC of the thyroid was classified as Bethesda 3 (atypia of undetermined significance). The cystic tumor was resected under total anaesthesia by the Sistrunk procedure (Fig. 3). The postoperative histopathological specimen consisted of a cystic tissue sample with the fibrous wall infiltrated by neutrophilic granulocytes, squamous cells and multiple foci of PTC. Total thyroidectomy with pyramidal lobe resection was performed 3 weeks after extirpation. Subsequent histopathological findings indicated nodular colloid goitre with absence of cervical lymph node metastases of the central neck compartment. The postoperative period was without complications. Hormonal suppression therapy was introduced immediately after the surgery. The patient has been examined once a year, including the evaluation of tumor markers (most recent thyroglobulin levels 0.32 μg/l) and a sonograph of the neck.

### 3.3 Results of case 3

**Fig. 4:**
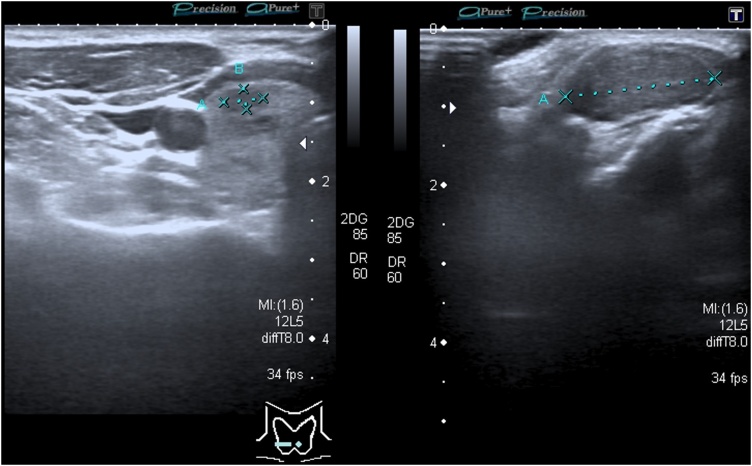
**Ultrasonography of the neck** solid hypoechogenic, slightly vascularised tissue 19 × 17 × 9 mm.

**Fig. 5:**
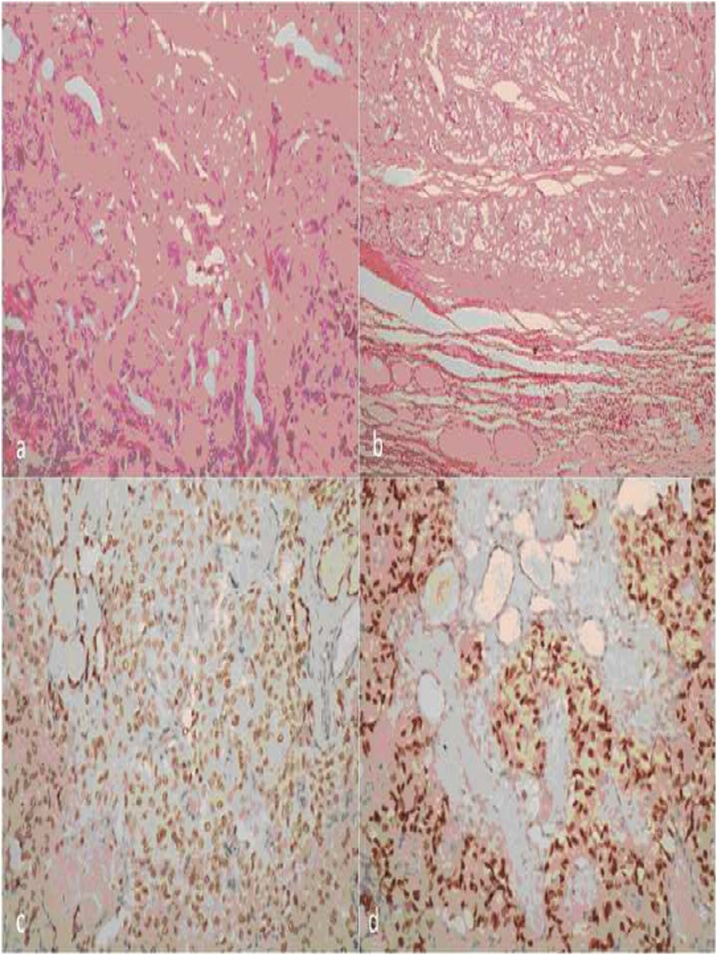
**Postoperative histopathology** a) H&E stained, magnification 200x, b) Border between tumor (top) and adjacent thyroid parenchyma (bottom; H&E stained, magnification 100x), c) thyroid transcription factor 1 (TTF-1) was immunohistochemically confirmed both in tumor cells and non-tumorous follicles within tumour (magnification 200x), d) Presence of estrogen receptors in tumor tissue, surrounded by non-tumorous thyroid parenchyma; immunohistochemical ER analysis (magnification 200×).

Clinical examination revealed a tumor of 20 × 20 × 10 mm and biochemical examination yielded values reference threshold. Ultrasonography detected a solid, hypoechogenic and slightly vascularised 19 × 17 × 9 mm tumor (Fig. 4), thyroid was of normal size and the parenchyma contained several lesions of various types (both hyperechogenic and hypoechogenic; >10 mm) and the cervical lymph nodes were not enlarged. Postoperative histopathological examination and immunophenotyping verified a 20 × 20 × 12 mm ovoid tumor in the separated, or aberrant thyroid tissue, typical for CMV-PTC. Papillae were lined with columnar cells, invasion into blood or lymph vessels was not detected. Presence of estrogen receptors was confirmed in the tumor tissue but not in the surrounding tissue (Fig. 5A–D). To exclude familial adenomatous polyposis (FAP), the patient was referred postoperatively for screening colonoscopy, which revealed a normal colon. A subsequent total thyroidectomy was performed 3 weeks later. Postoperative histopathology revealed colloid nodular goitre with prevalence of hyperplastic macrofollicular nodules without malignancy. After surgery, the patient underwent RAI. Oncological follow-up once every year, the patient is given 75 μg levothyroxine daily. The patient has had no sign of relapse for 5 years.

Possible causes of thyroid dysgenesis include the impact of maternal antithyroid immunoglobulins, genetic mutations, genes of transcription factors *TTF1, TTF2* and *PAX8*, and *HHEX* found in functional thyroid cells, but also in their precursors, which are essential for the early stages of thyroid morphogenesis [4,5].

Santangelo et al. reported other locations of ectopic thyroid in the head and neck regions, including the trachea, submandibular gland, maxilla, palatine tonsils, carotid bifurcation, the iris and the pituitary gland [4] with a clear female predilection of up to 7:1 [2]. In most cases, ectopic thyroid tissue is quantitatively deficient, resulting in an increased expression of TSH (thyroid-stimulating hormone), which causes hyperplasia of the ectopic tissue and its compensatory enlargement [2]. Evaluation of the differential diagnosis includes all lesions potentially arising in the region: lymphangioma, hemangioma, minor salivary gland tumors, lingual tonsil hypertrophy, midline branchial cysts, squamous cell carcinoma and lymphoma, dermoid cysts, lymphadenopathy, branchial cleft cysts, lipomas and sebaceous cysts [9]. With head or neck neoplasm, it is essential to exclude metastasis of PTC or other malignancies in separated or ectopic thyroid tissue [9]. Malignant transformation in lingual thyroid and thyroglossal duct cysts is rare, and the prevalence of differentiated thyroid carcinoma is less than 1 % of all cases. Its most common form is PTC or its histological subtypes (75–85 % cases) [5,9,10,18]. Thyroglossal duct carcinoma is often diagnosed incidentally during histopathological examination of a resected cyst [12,13]. It may be due to residual ectopic thyroid tissue in the duct (>90 % cases) or it may arise from the epithelium of the cyst wall [11,12]. CMV-PTC accounts for 0.2 % of all PTCs [14]. To date, 129 cases of CMV-PTC have been reported, with the female-to-male ratio 31:1 [15]. It can occur as a solitary tumor (sporadic form), or with FAP (usually a multifocal form), in approximately 39 % of all cases [16]. The papillae are lined with columnar cells, whose presence indicates a less favorable prognosis. CMV-PTC is more aggressive than conventional PTC, with more local recurrence and distant metastases [17]. In diagnosing ectopic thyroid tissue (including lingual thyroid), the most useful method is scintigraphy, single photon emission computed tomography (SPECT) with ^123^I-iodine or ^99m^Tc-, in combination with CT, or preferably a hybrid SPECT/CT as the most effective approach to exclude an eutopic thyroid gland and to localize ectopic thyroid tissue. Other imaging methods including ultrasound, MRI and FNAC, may help to further clarify the findings [4,9].

The future studies may be aimed at improving or streamlining the diagnosing procedure and at elucidation of the possible theories of thyroid dysgenesis.

## 4 Conclusion

Based on the successful treatment of our three patients and data from literature, we consider clinical examination combined with imaging methods (ultrasonography, and particularly CT scanning and scintigraphy), as the most important steps in the pre-operative diagnosis of thyroid ectopia. Ultrasonography should be used to exclude thyroid tissue in the normal localization and to distinguish other tumorous diseases. Benefits of FNAB in pre-operative diagnosing of ectopic thyroid are not favourable due to the difficult sampling and common non-diagnostic cytological findings, especially in the lingual thyroid tissue. In patients with clinical symptoms of globus pharyngeus, the most suitable therapy is surgical resection, followed by total thyroidectomy in the case of malignant transformation of the ectopic tissue. CMV-PTC is particularly rare, often arising in connection with FAP of the large intestine. Endoscopic examination of the large intestine is recommended to exclude FAP. Ectopic thyroid is a rare pathological condition, but it should be taken into consideration during differential diagnosis of tumorous lesions in the oropharynx and the neck.

## Funding

Supported by grants MH CZ – DRO (NHH, 00023884), IG192301, and CZ.02.1.01/0.0/0.0/16_013/0001674.

## Ethical approval

The present study and publication of data was approved by the Ethics Committee of Na Homolce Hospital, Prague (reference number for study 12/2018, reference number for publication 3.1.2019/1).

## Consent

All participants provided written informed consent to participate in the study.

## Author contribution

J. Lukas: devised the project and the main conceptual ideas, and wrote the majority of the manuscript.

D. Lukas: contributed to the implementation of the research, to the analysis of the results and to the writing of the manuscript.

I. Zemanova: contributed to the implementation of the research, the analysis of the results and to the writing of the manuscript.

A. Rulseh: contributed to the implementation of the research, the analysis of the results and to the writing of the manuscript.

J. Drabek: contributed to the analysis of the results and to the writing of the manuscript.

## Registration of research studies

Name of the registry: Research Registry.

Unique Identifying number or registration ID: 5191.

Hyperlink to the registration (must be publicly accessible): https://www.researchregistry.com/browse-the-registry#home/registrationdetails/5db0b9c3309ca70017e96ad2/.

## Guarantor

Professor Jiří Drábek.

## Provenance and peer review

Not commissioned, externally peer-reviewed.

## CRediT authorship contribution statement

**J. Lukáš:** Conceptualization, Data curation, Formal analysis, Funding acquisition, Investigation, Methodology, Project administration, Resources, Supervision, Validation, Visualization, Writing - original draft, Writing - review & editing. **J. Drábek:** Conceptualization, Formal analysis, Funding acquisition, Supervision, Writing - review & editing. **D. Lukáš:** Conceptualization, Data curation, Formal analysis, Investigation, Methodology, Project administration, Writing - review & editing. **I. Zemanová:** Conceptualization, Data curation, Formal analysis, Investigation, Methodology, Project administration, Validation, Writing - review & editing. **A. Rulseh:** Conceptualization, Data curation, Formal analysis, Investigation, Methodology, Project administration, Writing - original draft, Writing - review & editing.

## Declaration of Competing Interest

None.
